# Validity and reliability of the Modified Four Square Step Test in individuals with ankle sprain

**DOI:** 10.1186/s13018-024-04664-5

**Published:** 2024-03-15

**Authors:** Mehmet Yetiş, Hikmet Kocaman, Mehmet Canli, Halil Alkan, Hasan Yildirim, Nazim Tolgahan Yildiz, Şafak Kuzu

**Affiliations:** 1 Department of Orthopedics and Traumatology, Faculty of Medicine, Kirşehir Ahi Evran University, Kirşehir, Turkey; 2 School of Physical Therapy and Rehabilitation, Kirşehir Ahi Evran University, Kirşehir, Turkey; 3 Deparment of Physiotherapy and Rehabilitation, Faculty of Health Science, Muş Alparslan University, Muş, Turkey; 4 Department of Mathematics, Faculty of Kamil Özdağ Science, Karamanoğlu Mehmetbey University, Karaman, Turkey; 5 Department of Physiotherapy and Rehabilitation, Faculty of Health Sciences, Karamanoglu Mehmetbey University, Karaman, Turkey

**Keywords:** Ankle sprain, Modified Four Square Step Test, Validity, Reliability

## Abstract

**Background:**

Postural instability and gait abnormalities are frequently observed after an ankle sprain. A modified Four Square Step Test (mFSST) was developed to assess dynamic balance during gait. The aim of this study was to evaluate the reliability and validity of the mFSST in individuals with ankle sprains.

**Methods:**

The study included 39 individuals with grade 1 and 2 ankle sprains with a mean age of 30.36 ± 6.21 years. The dynamic balance of the participants was assessed with the mFSST and Timed Up & Go test (TUG). To determine the test-retest reliability of the mFSST, the test was repeated approximately 1 h apart.

**Results:**

The test-retest reliability of the mFSST was excellent (ICC = 0.85). Furthermore, when the concurrent validity of the mFSST was examined, a high correlation was found between with the TUG (*r* = 0.78, *p* < 0.001).

**Conclusion:**

The mFSST is a valid and reliable clinical assessment method for evaluating dynamic balance during walking in individuals with ankle sprains. We think that the mFSST is preferable in clinical evaluations because its platform is easy to prepare and requires very little equipment.

## Introduction

Ankle sprains are one of the most common musculoskeletal injuries [1–3]. Ankle sprains account for approximately 14% of all emergency hospital visits; moreover, ankle sprains are the subject of approximately 76.7% of studies examining ankle injuries [4]. In patients with ankle sprains, loss of range of motion, swelling, muscle weakness, and impaired postural control are observed in the acute phase [4, 5]. Studies have shown that impaired proprioception and neuromuscular control in individuals with ankle sprains can lead to re-injury and chronic ankle instability [6, 7]. In addition, pain and loss of function after a sprain negatively affect activities of daily living [8].

There are many measurement methods to be used in the clinic to evaluate postural control and balance in individuals with ankle sprains. Various measurements, such as Time Up & Go test (TUG), balance error scoring system, Star Excursion Balance Test (SEBT), and side-hop test, are used to evaluate postural stability and balance in individuals with ankle sprains [9–11]. While these tests provide a comprehensive evaluation of general dynamic balance, their specificity in assessing stepping balance in various directions is limited, which is a critical consideration in conditions such as ankle sprains. For instance, the SEBT primarily concentrates on multidirectional reaching with the foot, offering a less comprehensive assessment of stepping balance. Additionally, the time-consuming nature of performing the entire SEBT poses a practical challenge for clinicians [12]. Furthermore, the side-hop test, despite its performance-oriented nature, introduces a potential risk of re-injury when applied during the acute phase. Therefore, tests specifically designed to evaluate step-taking in diverse directions and dynamic balance aligned with an individual’s daily activities hold greater potential for individuals with ankle sprains [13]. The Modified Four Square Step Test (mFSST) is a performance test that assesses dynamic balance linked to various sensory and motor systems, including proprioception, vestibular, and visual systems [14]. The validity and reliability of mFSST were examined in individuals with stroke [14], Parkinson’s disease [15], multiple sclerosis [16], total knee arthroplasty [17], anterior cruciate ligament reconstruction [18], and older adults [19], and it was concluded that it is a valid and reliable measurement method for the assessment of dynamic balance. The most important advantage of mFSST over dynamic balance measurement methods is that it requires less equipment and is easy [14].

As it is known, in order for clinical assessment tests to be applied in a case group, their psychometric properties must be examined [20]. Examining the psychometric properties of the mFSST in individuals with ankle sprains will enable it to be used reliably in studies to be conducted in individuals with ankle sprains. Despite an extensive literature review, no prior study investigating the validity and reliability of the mFSST in individuals with ankle sprain was identified. Thus, the aim of our study was to examine the validity and reliability of the mFSST in individuals with ankle sprain.

## Methods

### Participants and setting

The study was conducted as a cross-sectional validity and reliability study with the inclusion of 39 individuals with ankle sprains. The participants were diagnosed with ankle sprain by an orthopedic and traumatologist, and the assessment tests were performed by a physiotherapist. Participants were evaluated in a room with a quiet and distraction-free flat floor. The duration of the tests was measured with the help of a stopwatch. The study was conducted in accordance with the Declaration of Helsinki. Before the evaluations, the participants were informed about the study, and their verbal and written informed consent was obtained. The ethics committee’s approval of the study was approved by Muş Alparslan University Scientific Research and Publication Ethics Committee (Date: 07.07.2023, Number: 07-2023/73).

The inclusion criteria were: (1) individuals with a first-time unilateral ankle sprain at least 3 months ago; (2) sedentary individuals with a metabolic equivalence (MET) level of < 600 METs per week according to the International Physical Activity Questionnaire; (3) individuals with ankle sprain diagnosed by an orthopedic and traumatology specialist with grade 1–2 severity; (4) individuals who volunteered to participate in the study. Subjects with ankle fracture, other neuromuscular pathology, ankle dislocation, grade 3 ankle sprain, and recurrent ankle sprain were excluded from the study. The study conducted by Lexell and Downham was used as a reference for the calculation of the sample size of the study [21]. According to the results of this study, it was stated that the sample size should consist of 30–50 participants in reliability studies of clinical assessment tests.

### Data collection

Sociodemographic characteristics of the participants (age, body mass index-BMI, injury history, history of ankle sprain, and injured extremity) were recorded before the study. Participants were examined by an orthopedic and traumatology specialist with 20 years of experience in the field (MY). To avoid inter-rater bias, all clinical assessment tests were performed by a physiotherapist specialized in orthopedic rehabilitation (MC). In the first evaluation, mFSST was performed, and then the TUG test was evaluated after an adequate rest period (five minutes) was provided. One hour later, after the first assessment, mFSST was re-evaluated by the same physiotherapist in all patients.

### Modified Four Square Step Test (mFSST)

The mFSST is a clinical test that assesses dynamic balance while stepping forward, backward, and sideways. The test was performed on a floor divided into four square, which was formed by gluing two one-meter-long tapes perpendicular to each other. These four squares were numbered clockwise from 1 to 4 respectively. At the beginning of the test, participants stood in square 1, facing square 2. Participants were then asked to step forward, to the right, backward, and to the left as quickly as possible, starting with their self-selected foot (2-3-4-1, respectively). Then, they were asked to step counterclockwise to the right side, forward, left side, and backward and come back to square 1 (4-3-2-1, respectively). The test protocol was explained to the participants before the test. Two trial tests were conducted to ensure that the participants understood the test. The test was conducted once to avoid fatigue of the participants. The completion time of the test was recorded in seconds. In cases of loss of balance or touching the bands during the test, the test was repeated after 5 min [14].

### Timed Up & Go Test (TUG)

The TUG was developed to assess dynamic balance and mobility. For the test, the participants were asked to stand up from the chair without support with their hands, walk 3 m, and then walk back to the chair without support and sit down again. The stopwatch was started when the participants stood up from the chair and stopped when they returned to the chair and sat down again. The test was repeated three times, and the average time was recorded in seconds [22].

### Statistical analysis

The statistical analysis of the study was designed to cover descriptive statistics, correlation and reliability analyses. The descriptive statistics were reported via arithmetic mean, standard deviation and min-max results for quantitative data (age, BMI, etc.) and frequency and percentage values for qualitative data (gender, grade, etc.). The relationship between TUG and mFSST for both test and retest measurements was assessed with Spearman’s correlation coefficient. The intra-class correlation coefficient (ICC) was utilized to determine the level of agreement between test and retest measurements in the mFSST.

Additionally, the standard error of measurement_95_ (SEM_95_) and the minimal detectable change_95_ (MDC_95_) were evaluated to determine the level of error between measurements (i.e. to measure the stability of the measurements) and to detect the minimal change that falls outside the measurement error, respectively. ICC, the SEM_95_ and the MDC_95_ score values were calculated at 95% confidence level to assess the reliability and consistency between test and retest measurements of the mFSST. The SEM_95_ and MDC_95_ scores were calculated using the formulas given below:

$${\rm{SE}}{{\rm{M}}_{{\rm{95}}}}\,{\rm{ = }}\,{\rm{SD}}\, \times \,\surd \left( {{\rm{1}}\, - \,{\rm{ICC}}} \right)$$

$${\rm{MD}}{{\rm{C}}_{{\rm{95}}}}\,{\rm{ = }}\,{\rm{1}}{\rm{.96}}\, \times \,{\rm{SE}}{{\rm{M}}_{95}}\, \times \,\surd 2$$

where corresponds to the standard deviation based on the difference values of measurements. Regarding SEM_95_ and MDC_95_ scores, lower values indicate more consistent and generalizable results. The ICC coefficient is interpreted as good for [0.60–0.80] range and excellent for [0.80-1.0] range [23]. In order to confirm the reliability between measurements, Bland-Altman and violin plots representing the dispersion of test-retest measurements are separately presented.

Throughout the study, the assumption of normality, which is one of the assumptions of parametric test (e.g. Pearson coefficient), was checked by Shapiro-Wilk test and the assumption of linearity via scatter plot. The significance level was set as fixed at 0.05. The results were derived by using R (version 4.3.2) and MedCalc (version 22) softwares.

## Results

Demographic and physical characteristics of 39 individuals with ankle sprains included in the study are given in Table 1.

**Table 1:** Descriptive statistics of demographic variables (*n* = 39)

	Mean	SD	Minimum	Maximum
Age (years)	30.36	6.21	19	44
BMI (kg/m^2^)	23.70	3.30	21	27
Duration of injury (week)	15.71	4.12	12	21
		**Count**	**Percentage (%)**	
Gender	Male	22	56.4	
	Female	17	43.6	
Dominant lower extremity	Right	29	74.4	
	Left	10	25.6	
Injured ankle side	Right	22	56.4	
	Left	17	43.6	
Injury level	Grade 1	22	56.4	
	Grade 2	17	43.6	

SD: Standart deviation, BMI: Body mass index

In the second part of the study, the relationship between the mFSST score and the TUG score for test measurements was analyzed via Spearman correlation analysis and given in Table 2. The interpretation reference is given as follow [24]: (i) low for 0.05 < *r* < 0.40, (ii) moderate for 0.40 < *r* < 0.70, (iii) high for 0.70 < *r* < 1.00. The test (*r* = 0.78) and retest (*r* = 0.69) measures of the mFSST were both positively, high and statistically significantly correlated with the TUG (*p* < 0.001).

**Table 2:** The relationships between TUG and mFSST for both test and retest measurements

		mFSST (Test)	mFSST (Retest)
TUG (sec)	r	0.78	0.69
	p	< 0.001	< 0.001

*p* < 0.001, r: Spearman’s correlation coefficient, mFSST: Modified Four Square Step Test, TUG: Timed Up & Go test

The ICC, SEM_95_ and MDC_95_ scores of the mFSST scores are provided in Table 3 in summary. According to these results, the ICC score indicates that there is excellent consistency (reliability) between the test and retests measurements, while the SEM_95_ and MDC_95_ scores are reasonable and adequate.

**Table 3:** The intra-class correlation coefficient and confidence interval range values from mFSST

	Measurement	Mean (SD)	ICC	Confidence Interval of ICC (95%)		SEM_95_	MDC_95_
				Lower Bound	Upper Bound		
mFSST (sec)	Test	13.40 (2.05)	0.85	0.73	0.92	0.42	1.16
	Retest	13.68 (2.03)					

mFSST: Modified Four Square Step Test, ICC: Intra-class correlation coefficient, SEM_95_: Standard error of measurement, MDC_95_: Minimal detectable change

**Fig. 1:**
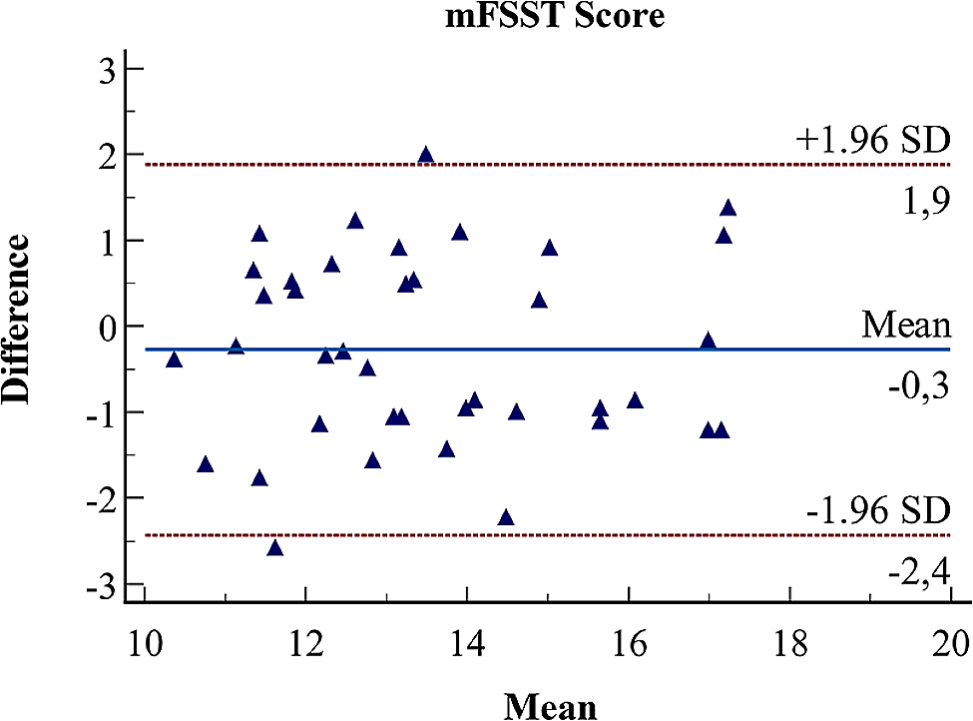
The Bland-Altman plot based on the test and retest measurements for the mFSST

**Fig. 2:**
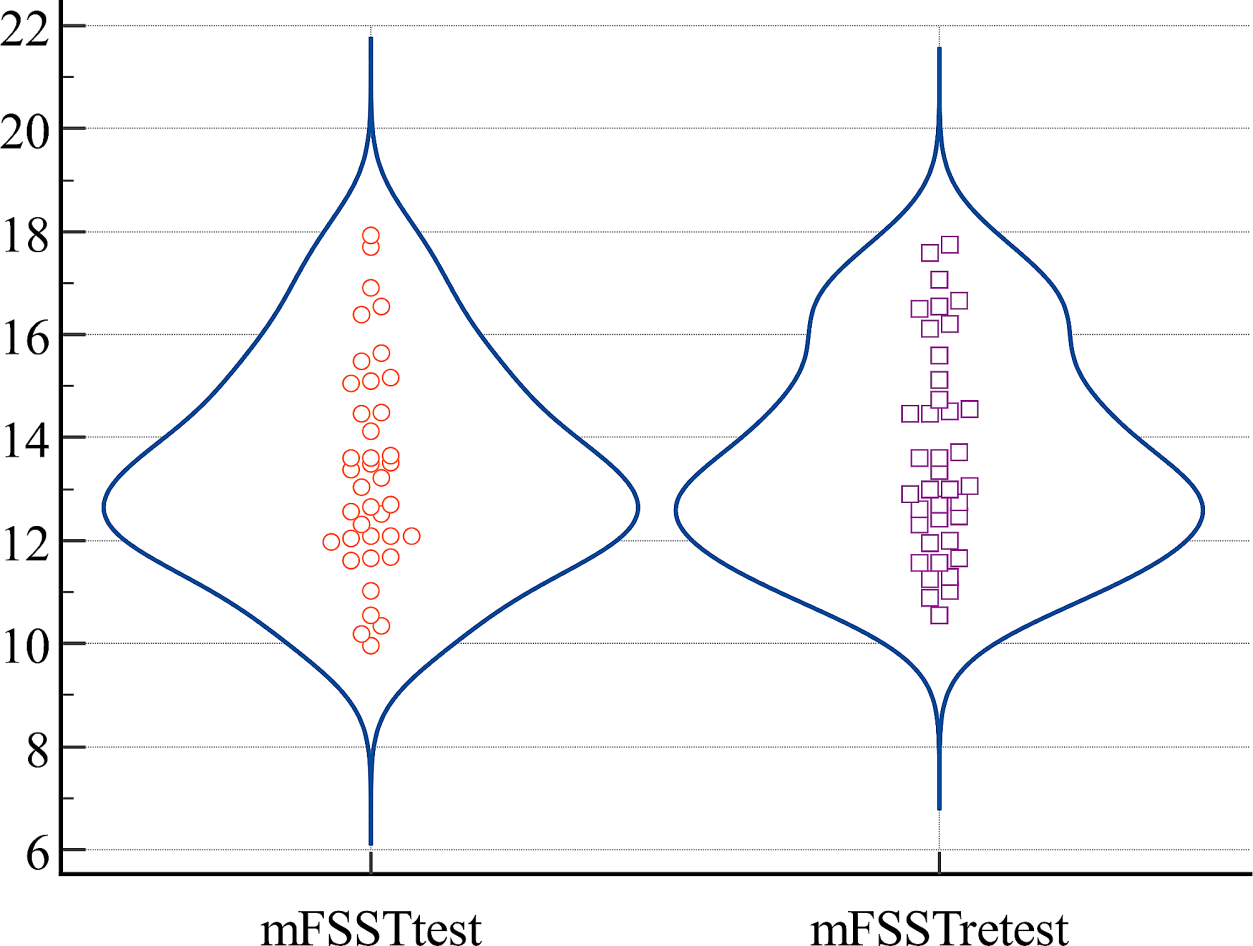
The violin plot of test and retest measurements for the mFSST

For more insight and to support the reliability of the measurements, a Bland-Altman plot of the test and retest measurements was obtained and presented in Fig. 1. According to this figure, the test difference scores are within the 95% confidence interval and show a spread around zero. Furthermore, the violin plot was obtained and given in Fig. 2 in order to have a clearer understanding of the distribution of the rater’s scores. Figure 2 indicates that the test scores are quite close to each other, consistent and more stable, and no unusual changes are detected.

## Discussion

Previous studies have concluded that the Four Square Step Test (FSST) is valid and reliable in dynamic balance assessment. The mFSST, which was developed as a modified version of the FSST, was made safer and easier in clinical evaluations by using a band instead of a cane. According to the results of the present study, the test-retest reliability of the mFSST is excellent in individuals with ankle sprains and that it is a valid clinical assessment method when compared with the TUG.

Research indicates that a minimum of 73% of individuals experiencing ankle sprains develop persistent symptoms such as pain, sensations of instability (giving way), impaired proprioception and neuromuscular control. These residual symptoms elevate the risk of re-injury and contribute to the development of chronic ankle instability [25–27]. Studies have reported that mechanical ankle instability following an ankle sprain is not fully recovered within the initial 6–12 weeks post-injury [28]. A substantial portion of patients continue to exhibit mechanical laxity and report instability, reduced function, pain, and/or swelling for up to one year after the initial injury. Despite improvements in initial symptoms, individuals with an ankle sprain should undergo comprehensive rehabilitation. Evidence suggests that inclusive rehabilitation programs, encompassing proprioceptive, neuromuscular control, and balance training, can significantly diminish the risk of recurrent ankle sprains [25, 28].

Measurement tools such as SEBT, Single-Leg Hop Test, Biodex Balance System, and Y-balance test are available to evaluate the postural balance of individuals with ankle sprains [25, 29, 30]. However, unlike other measurement tools, the platform of the mFSST can be easily prepared and requires very little equipment.

The test-retest reliability of the mFSST has been examined in different populations. This test was found to be a reliable measurement method in patient groups such as Parkinson’s (ICC = 0.96–0.99) [15], multiple sclerosis (ICC = 0.99) [16], stroke (ICC = 0.94) [14], elderly (ICC = 0.98) [19], ACL reconstruction (ICC = 0.92) [18], and total knee arthroplasty (ICC = 0.97) [17]. In our study, similar to other populations, we found excellent test-retest reliability of the mFSST in individuals with ankle sprains (ICC = 0.85). The mFSST was performed in 13.4 s on individuals with ankle sprains. The test execution time was 18.4 s in the stroke group [14], 13.7 s in the elderly group [19], 11.4 s in the group with multiple sclerosis [16], 9.01 s in the group with Parkinson’s disease [15], 7.6 s in the group with ACL reconstruction [18], and 12.05 s in the group with total knee arthroplasty [17].

Concurrent validity of the mFSST was determined using the TUG test. The choice of the TUG test in our study was deliberate for several reasons: first, the SEBT lacks a time-based component [12]; second, the side-hop test concentrates on physical performance [13]; and third, the TUG test is commonly employed for assessing concurrent validity in other studies evaluating the validity and reliability of the mFSST [14, 16, 18, 19]. This selection ensures meaningful comparisons of our findings with existing literature. In our study, there was a strong correlation between mFSST and TUG (*r* = 0.78). In other populations, correlation coefficients between mFSST and TUG were 0.72 in individuals with stroke [14], 0.78 in individuals with multiple sclerosis [16], 0.75 in elderly individuals [19] and 0.76 in individuals with ACL-reconstruction [18]. The concurrent validity of the mFSST was assessed in stroke individuals using the Berg Balance Score (BBS), Activities-Specific Balance Confidence Scale (ABC), and FSST. The study found a strong relationship between mFSST and all outcome measures except ABC [14]. Another study found a strong association between mFSST and BBS in the acl-reconstructed population [18]. The reason why we did not use outcome measures such as BBS and ABC in this study is that we think that patient-reported outcome measures would not provide objective results.

As a result of the study, the SEM_95_ value of the mFSST was 0.42 and the MDC_95_ value was 1.16. While the SEM_95_ value represents the margin of error that may occur in clinical measurement tests, the MDC_95_ value is important as it represents the minimum variation between clinical measurements. The SEM_95_ value of mFSST was 0.41 and MDC_95_ value was 1.13 in individuals with multiple sclerosis [16]; the SEM_95_ value was 2.21 and MDC_95_ value was 6.12 in the elderly [19]; the SEM_95_ value was 0.15 and MDC_95_ value was 0.41 in individuals with ACL reconstruction [18]; and SEM_95_ value was 1.11 in individuals with total knee arthroplasty [17]. SEM_95_ and MDC_95_ of mFSST in other populations were not calculated.

Some limitations of this study can be mentioned. The first of these is that it may be valuable to prefer a one-week period between test-retest measurements instead of approximately one hour to reduce the effect of participants learning the test. A second limitation is that the reliability of the test was evaluated by a single rater. Another point is that the order of the steps between the squares during the test should be well explained to the participants and demonstrated practically. Because the steps between the squares may be confusing and may cause a loss of time since it is a time-based test. In future studies, test-retest reliability should be evaluated by a different rater.

## Conclusion

The test-retest reliability of the mFSST in individuals with ankle sprain is excellent and its concurrent validity is strong. Since the test platform is easy to prepare and requires very little equipment, it can be preferred for dynamic balance assessment of individuals with ankle sprain.

## Data Availability

The datasets analyzed during the current study are available from the corresponding author on reasonable request.

## References

[1] Fong DTP, Hong Y, Chan LK (2007). A systematic review on ankle injury and ankle sprain in sports. Sports Med.

[2] Candeniz Ş, Kocaman H, Çelik SE et al. Cross-cultural adaptation, reliability, and validity of the Turkish version of the Cumberland Ankle Instability Tool. Musculoskel Sci Prac. 2023;68(2023):1–6. 10.1016/j.msksp.2023.102873 37897935

[3] de Azevedo Sodré Silva A, Sassi LB, Martins TB (2023). Epidemiology of injuries in young volleyball athletes: a systematic review. J Orthop Surg Res.

[4] Fong DTP, Man CY, Yung PSH (2008). Sport-related ankle injuries attending an accident and emergency department. Injury.

[5] Youdas JW, McLean TJ, Krause DA (2009). Changes in active ankle dorsiflexion range of motion after acute inversion ankle sprain. J Sport Rehabil.

[6] Hintermann B, Boss A, Schäfer D (2002). Arthroscopic findings in patients with chronic ankle instability. Am J Sport Med.

[7] Ferran NA, Oliva F, Maffulli N (2009). Ankle instability. Sports Med Arthrosc Rev.

[8] Ivins D (2006). Acute ankle sprain: an update. Am Fam Physician.

[9] Arnold BL, De La Motte S, Linens S (2009). Ankle instability is associated with balance impairments: a meta-analysis. Med Sci Sports Exerc.

[10] Hertel J, Braham RA, Hale SA (2006). Simplifying the star excursion balance test: analyses of subjects with and without chronic ankle instability. J Orthop Sport Phys.

[11] Docherty CL, Arnold BL, Gansneder BM (2005). Functional-performance deficits in volunteers with functional ankle instability. J Athl Train.

[12] Coughlan GF, Fullam K, Delahunt E (2012). A comparison between performance on selected directions of the star excursion balance test and the Y balance test. J Athl Train.

[13] Yoshida M, Taniguchi K, Katayose M (2011). Analysis of muscle activity and ankle joint movement during the side-hop test. J Strength Conditioning Res.

[14] Roos MA, Reisman DS, Hicks GE (2016). Development of the modified four square step test and its reliability and validity in people with stroke. JRRD.

[15] Boddy A, Mitchell K, Ellison J (2023). Reliability and validity of modified Four Square Step Test (mFSST) performance in individuals with Parkinson’s disease. Physiother Theor Pr.

[16] Özkeskin M, Özden F, Ar E (2023). The reliability and validity of the 30-second chair stand test and modified four square step test in persons with multiple sclerosis. Physiother Theor Pr.

[17] Unver B, Sevik K, Yarar HA (2021). Reliability of the Modified Four Square Step Test (mFSST) in patients with primary total knee arthroplasty. Physiother Theor Pr.

[18] Kocaman H, Canlı M, Alkan H (2023). The reliability and validity of the Modified Four Square Step Test in individuals with Anterior Cruciate Ligament Reconstruction. Indian J Orthop.

[19] Özkeskin M, Özden F, Tuna S (2021). The reliability and validity of modified four-square-step-test and Step-Test in older adults. Phys Occup Ther Geri.

[20] Mokkink LB, Prinsen CAC, Bouter LM (2016). The Consensus-based standards for the selection of health measurement INstruments (COSMIN) and how to select an outcome measurement instrument. Braz J Phys Ther.

[21] Lexell JE, Downham DY (2005). How to assess the reliability of measurements in rehabilitation. Am J Phys Med Rehab.

[22] Coelho-Junior HJ, Rodrigues B, de Oliveira Gonçalves I (2018). The physical capabilities underlying timed up and go test are time-dependent in community-dwelling older women. Exp Gerontol.

[23] Koo TK, Li MY (2016). A guideline of selecting and reporting intraclass correlation coefficients for reliability research. J Chiropr Med.

[24] Schober P, Boer C, Schwarte LA (2018). Correlation coefficients: appropriate use and interpretation. Anesth Analg.

[25] Alghadir AH, Iqbal ZA, Iqbal A (2020). Effect of chronic ankle sprain on pain, range of motion, proprioception, and balance among athletes. Int J Env Res Pub He.

[26] Ferran NA, Maffulli N (2006). Epidemiology of sprains of the lateral ankle ligament complex. Foot Ankle Clin.

[27] Bridgman SA, Clement D, Downing A (2003). Population based epidemiology of ankle sprains attending accident and emergency units in the West Midlands of England, and a survey of UK practice for severe ankle sprains. Emerg Med J.

[28] Safran MR, Benedetti RS, Bartolozzi AR (1999). Lateral ankle sprains: a comprehensive review: part 1: etiology, pathoanatomy, histopathogenesis, and diagnosis. Med Sci Sports Exerc.

[29] Ko J, Rosen AB, Brown CN (2018). Functional performance tests identify lateral ankle sprain risk: a prospective pilot study in adolescent soccer players. Scand J Med Sci Spor.

[30] Lim C (2017). Short-term effect of spiral taping on the pain and walking performance of individuals with chronic ankle instability. J Phys Ther Sci.

